# Integrin Inhibitors in Prostate Cancer

**DOI:** 10.3390/cancers10020044

**Published:** 2018-02-06

**Authors:** Maylein C. Juan-Rivera, Magaly Martínez-Ferrer

**Affiliations:** 1 Department of Pharmaceutical Sciences, School of Pharmacy, University of Puerto Rico, Medical Sciences Campus, San Juan, PR 00936, USA; maylein.juan@upr.edu; 2 University of Puerto Rico Comprehensive Cancer Center, Medical Sciences Campus, San Juan, PR 00936, USA

**Keywords:** *Gleditsia sinensis*, d-pinitol, Abituzumab, d-aminoacid, Contortrostatin, α2β1, αvβ3, metastasis, PC3, 22RV1

## Abstract

Prostate cancer (PCa) is the most frequently diagnosed cancer and the third highest cause of cancer-related deaths in men in the U.S. The development of chemotherapeutic agents that can bind PCa tumor cells with high specificity is critical in order to increase treatment effectiveness. Integrin receptors and their corresponding ligands have different expression patterns in PCa cells. They have been identified as promising targets to inhibit pathways involved in PCa progression. Currently, several compounds have proven to target specific integrins and their subunits in PCa cells. In this article, we review the role of integrins inhibitors in PCa and their potential as therapeutic targets for PCa treatments. We have discussed the following: natural compounds, monoclonal antibodies, statins, campothecins analog, aptamers, d-aminoacid, and snake venom. Recent studies have shown that their mechanisms of action result in decrease cell migration, cell invasion, cell proliferation, and metastasis of PCa cells.

## 1. Introduction

Integrins are transmembrane protein receptors that attach cells to the extracellular matrix (ECM) or that bind ligands secreted by other cells [1,2]. They are composed of two distinct α and β subunits. Metal ions are required to form non-covalent associations between these subunits. There are 18 α and 8 β subunits that can assort to form 24 distinct integrins, which are responsible for interaction with ligands such as laminin, fibronectin, vitronectin, and collagen (Table 1) [1]. Integrins are classified as type I membrane proteins and have been shown to play important roles in multiple cell activities, including cell signaling networks, cell growth, cell differentiation, cell mobility, and cell survival [1,2]. In addition, they are normally expressed in an inactive state and can be activated through inside-out or outside-in mechanisms. Each integrin can bind specific ligands recognizing the RGD sequence (the tripeptide Arg-Gly-Asp) present in ECM proteins. Interactions between integrins and the ECM induces cluster formation with other signaling molecules such as focal adhesions kinase (FAK) molecules, which result in cell adhesion and cell migration [1,3,4]. Changes in integrin gene expression have been significantly associated with many types of malignancies, including prostate cancer (PCa) [1,3,4].

### Integrin Alterations and Prostate Cancer

The Surveillance, Epidemiology, and End Results (SEER) Program of the National Cancer Institute have estimated there will be 161,360 new cases of PCa in 2017 [5]. Currently, the clinically available treatments for PCa include: androgen deprivation, prostatectomy, and radiation therapy. However, if the tumor is at advanced stages, most of these therapies are unsuccessful [1,2,3,4]. Therefore, the development of additional therapeutic strategies for individuals diagnosed at an advanced stage has become a major goal in the field of PCa therapeutics. Due to the multistep nature of prostate carcinogenesis, treatment comprised of a combination of inhibitory compounds is thought to be necessary to effectively limit PCa progression. Integrins are now considered promising targets for PCa therapeutics because of their role in modulating cell adhesion and migration in PCa, and promoting intracellular trafficking, which results in cell proliferation, cell invasion, tumor growth, neoangiogenesis, and metastasis [1,3,4]. Integrin inhibitors downregulate the expression of growth factor receptors that promotes tumor growth and cell proliferation (e.g., insulin-like growth factor: IGF-IR) or mutations in tumor suppressor genes that regulates cell migration on integrins (e.g., Phosphatase and tensin homolog: PTEN), among others [1,4]. In this review, specific therapies will be discussed and we give an overview of the evidence supporting how changes in the expression and function of integrins contribute to prostate carcinogenesis and are therefore promising targets for the development of PCa treatments.

## 2. Natural Herbs Inhibit α2β1 and αvβ3 Integrins

The α2β1 integrin is a well-known receptor for collagen, which is highly expressed in PCa and its activation/phosphorylation has been implicated in PCa progression. This integrin is the dominant cell-surface protein that has the most relevant role in the invasion of PC3 PCa cells when compared to other integrins [1,2]. The αvβ3 integrin has been reported to contribute to PCa progression by promoting angiogenesis, survival, and invasion [1,2]. For this reason, the development of novel therapeutics agents that can modulate the function of the α2β1 and αvβ3 integrins may be effective to inhibit PCa metastasis.

### 2.1. Gleditsia Sinensis (GS)

*Gleditsia sinensis* (GS) is a natural herb that has been used in oriental medicine to treat many diseases, such as carbuncles, suppuration, obesity, and swelling [2]. Its therapeutic properties are found in different parts of the plant, including the seeds, fruits, and cortex. There is evidence supporting the antitumor activity of *G. sinensis* against different types of cancer, such as gastric cancer, lung cancer, colon cancer, and PCa [2]. *G. sinensis* extract has shown to affect α2β1 integrin expression, and to have regulatory effects in cell migration and adhesion in PCa cells. Recent studies have shown that the administration of non-toxic levels of water extracted from *G. sinensis* (50 μg/mL) significantly inhibited collagen-mediated cell migration and cell adhesion in PC3 cells through the inactivation of the expression of the α2β1 integrin [2]. Interestingly, administration of *G. sinensis* extract specifically inhibits the expression of the α2 subunit (not the β1 subunit) [2]. Inhibition of α2β1 integrin expression results in the reduction of FAK activation/phosphorylation levels during the process of cell adhesion to collagen [3,4,5,6,7,8,9]. In this pathway, FAK becomes autophosphorylated (pFAK-Y397) and subsequently activates Src family kinases and other related signaling pathways including focal adhesion to the nucleus, which regulates cell migration and cell invasion [1,2,3,4,5,6,9]. Cells with high levels of activated FAK exhibit increased migration, whereas cells with low levels of activated FAK exhibit decreased migration [1,6,7,8,9].

*G. sinensis*-mediated inhibition of cell migration and adhesion was shown to be more effective in controlling the progression of late stages in metastatic prostate tumors compared to early stage PCa. Furthermore, a study using an in vivo xenograft tumor model showed that mice treated with a 200 μg/mL oral dose of *G. sinensis* extract developed significantly smaller tumors compared to non-treated, nude mice [2]. Ryu et al.’s study supported the antitumor effect of water extracted from *G. sinensis* in vivo when they observed that treatment with high concentrations (>50 μg/mL) resulted in DNA fragmentation and induction of programmed cell death [2]. This was the first evidence demonstrating that *G. sinensis* extract inhibits collagen, which is a mediator of cell migration and cell adhesion in PC3 PCa cell line via the inactivation of the α2β1 integrin.

### 2.2. d-Pinitol

d-pinitol is a phytochemical that was identified as the active and main ingredient in soy-based foods and legumes [9,10]. Mature and dried soybean seeds contain up to 1% d-pinitol. This phytochemical functions as an osmolyte in plants by improving tolerance to drought or heat stress [9]. In terms of bioactivity, this compound possesses multifunctional properties, including acting as a stimulatory, anti-inflammatory, cardioprotective, and antihyperlipidemic compound, in addition to contributing to creatine retention [10,11,12]. Recent studies have shown its potential as a chemotherapeutic agent against lung, bladder, and breast cancer [9].

d-pinitol has been shown to inhibit cell migration and invasion at non-cytotoxic concentrations (0 μM to 30 μM) in the PC3 and DU145 androgen-independent PCa cell lines [9]. Also, it has been shown that d-pinitol reduces αvβ3 integrin mRNA expression, resulting in the inhibition of metastasis [9]. d-pinitol downregulates αvβ3 integrin expression through two important pathways. First, this compound inhibits the FAK/c-Src kinase phosphorylation pathway, which plays an important role in cell motility and invasion in a dose-dependent manner [9,13]. Second, d-pinitol decreases p65 phosphorylation in the NF-κB signal transduction pathway [9]. This pathway regulates cell migration and metastasis [9]. Together, these findings support that the anti-metastasis activity of d-pinitol in PCa cells is mediated through the modulation of αvβ3 integrin cell surface expression.

## 3. Monoclonal Antibodies Inhibit αv and β1 Integrin

Integrins that contain the αv subunit contribute too many cellular functions that have been shown to promote malignancies, such as melanoma, renal cancer, colorectal cancer, and PCa [13]. Inhibition of αv integrin activation has been shown to reduce cell survival and induce cell cycle blockade [13]. This study also showed that this also reduced tumor growth and metastasis, thereby providing the desired antitumor effect. These findings have served as the foundation for the development of clinically viable methods to target the integrin αv subunits.

On the other hand, β1 integrins are highly expressed in the basal cell layer and are localized in the basal cell/stromal interface, where integrins interact with the ECM [13]. The activation of β1 integrins play a critical role in PCa metastasis potential by increasing their resistance to cell death [14]. Nevertheless, the mechanism by which β1 integrins are activated in PCa cells is yet to be elucidated [14]; however, it is important to mention that β1 integrins are expressed in 65% of PCa tumors [14].

### 3.1. mAb Abituzumab (DI17E6, EMD, 525797)

Monoclonal antibodies (mAbs) represent the largest class of therapeutic agents that specifically recognize cell surface antigens [13,14,15]. To date, mAbs have been approved by the Food and Drug Administration (FDA) and are currently used in oncology as diagnostic and treatment tools [14,15]. Abituzumab, a monoclonal antibody (mAb), was developed to target the integrin αv subunits [13]. Abituzumab’s mechanism of action is based on the recognition of the αv integrin extracellular domains, which inhibit the binding of ligands to αv heterodimers (αvβ1, αvβ3, αvβ5, αvβ6, and αvβ8) without cross-reacting with other members of the integrin family [13]. The ability of abituzumab to target αv subunits results in the suppression of PCa metastasis through the inhibition of cell-to-cell interactions, cell-to-ECM interactions, cellular invasion, cell migration, and cell signaling [13]. Abituzumab suppresses the activation of multiple integrin signaling pathways, such as FAK, AKT, and ERK, which have been shown to promote cell growth, cell motility, cell invasion, and tumor growth [1,2,4,5,9,13]. In addition, results from a phase I trial in PCa patients with bone metastasis showed potential antitumor activity [4]. These findings provide the rationale for future studies to validate this drug to control integrin-mediated PCa progression and to develop additional of αv integrin inhibitors to treat this malignancy.

### 3.2. mAb 33B6

The 33B6 mAb was developed as an inhibitor of the activated conformation of β1 integrin [14]. Lee et al. studied the activated β1 integrin using autophosphorylation of the FAK (pFAK-Y397) pathway in the PC3-mm2 PCa cell line [2,4,5,9,14]. The 33B6 mAb was shown to significantly inhibit ECM-mediated binding affinity, cells spreading on collagen, cell migration (10 to 20%), apoptosis (3%), and cell survival in PC3-mm2 cells [14]. Interestingly, treating this cell line with the 33B6 mAb led to the inhibition of β1 integrin through the FAK and AKT phosphorylation pathways [14]. These findings confirm that targeting the activated conformation of β1 integrin results in the prevention of PCa metastatic progression [1,2,4,5,9,13,14].

## 4. Statins and Campothecins Analogs Inhibits αvβ3 Integrins

The αvβ3 integrin is highly expressed in invasive and metastatic PCa cells and can bind ligands such as fibrinogen [16]. Recent studies have shown that the use of statins can inhibit the activity of αvβ3 integrin in PCa [16]. Statins are lipid lowering drugs with low molecular weight that inhibit HMG-CoA reductase. This enzyme is a component of the cholesterol synthesis pathway, which regulates various cell functions [16,17]. Previous studies have shown that while statins protect the vasculature via the activation of endothelial cells, they also possess anticancer properties by inhibiting proliferation and micrometastasis of malignant cells [16,17].

### 4.1. Simvastatin

Studies are currently validating the use of statins for PCa treatment [16,17]. Interestingly, a recent clinical study reported a 45% reduction in PCa recurrence after radical prostatectomy in patients taking statins, such as simvastatin [16]. The anticancer efficacy of simvastatin has been studied in LNCap and PC3 PCa cell lines [16]. Simvastatin inhibits αvβ3 integrin and the AKT (a serine-threonine kinase) pathway, which plays a role by reducing micrometastasis [16,17]. Also, this drug induces apoptosis via the modulation of cell survival and the activation of the extrinsic apoptotic pathway, including caspase-8, caspase-3, and caspase-9 [16,17].

The ability of statins to control tumor cells and their microenvironment could be useful in improving the efficacy of the currently available chemotherapeutic drugs when using a combination chemotherapeutic approach [16,18]. Simvastatin treatment has been shown to impair inside-out activation of the αvβ3 integrin and to reduce their affinity for specific ligands, such as fibrinogen [16]. Furthermore, co-treatment of PC3 cells with simvastatin and AP7.4 (αvβ3 integrin activating antibodies) rescued the impaired micrometastasis, thereby confirming that simvastatin has a direct effect on αvβ3 integrin by reducing its ligand affinity [16,18,19,20].

### 4.2. 10-Hydroxycamptothecin (10-HCPT)

Scientists are striving to develop new anticancer compounds partly because conventional cancer drugs have limited specificity and bioavailability [21]. The development of the campothecin analog 10-hydroxycamptothecin (10-HCPT) and its encapsulation in a polyamidoamine (PAMAM) polymer represent an advantage to selectively target PCa cells overexpressing the αvβ3 integrin through high affinity interactions [21,22]. This drug carrier has the ability to overcome problems associated with drug leakage in circulation and nonspecific effects on normal tissues [21]. The encapsulation of 10-HCPT in PAMAM results in high loading efficiency, high stability, and high water solubility without drug leakage during treatment in PCa cells [21]. The delivery system occurs by covalent conjugation stabilizing 10-HCPT inside the PAMAM surface and non-covalent complexation that keeps the compound in its inactive form until its release under specific target conditions [21,22].

Kong et al. investigated the antitumor activity of 10-HCPT in 22RV1 androgen-dependent PCa cells, which have high αvβ3 integrin expression, and in MCF-7 breast cancer cells, which express low levels of αvβ3 integrin [21]. The mechanism of action of 10-HCPT consists in inhibiting DNA topoisomerase I, an enzyme that is a ubiquitously overexpressed in a variety of tumor cell lines and is involved in DNA regulation during cell replication, recombination, and transcription [21]. 10-HCPT encapsulation has demonstrated anticancer activity against 22RV1 PCa cells with much lower cytotoxicity activity against MCF-7 breast cancer cells through high affinity interactions [21]. The anticancer effect of 10-HCPT is internalized with selectivity into PCa cells via receptor-mediated endocytosis. In comparison with free 10-HCPT, the 10-HCPT encapsulated in PAMAM exhibits very high cytotoxicity in PCa cells expressing high levels of αvβ3 integrin, demonstrating good specificity [21,22].

## 5. Aptamers Inhibits α6β4 Integrins

The α6β4 integrin belongs to the group of laminin-332 binding integrins that are present in endothelial, epithelial, and Schwann cells, as well as in keratinocytes [23,24]. The α6β4 integrin is the central component of hemidesmosomes, which mediate cell adhesion by connecting the intracellular keratin cytoskeleton to the cell basement membrane [23]. Nevertheless, the β4 integrin has distinctive cytoskeletal and signaling functions via its 1017 amino acid long cytoplasmic domain [23]. This domain can be phosphorylated by protein kinase C or by interactions with growth factor receptors that result in the release of the α6β4 integrin from hemidesmosomes [23]. The phosphorylation of the α6β4 integrin promotes migration and pre-metastasis signaling pathways [23]. Recent studies with different types of cancer, such as lung cancer and colon adenocarcinoma, have shown that the inhibition of the α6β4 integrin/laminin-332 interaction reduces cell growth, invasion, and metastasis [23,24,25,26,27,28,29].

### Integrin α6β4-Specific DNA Aptamer

Aptamers, are short single stranded oligonucleotides with high affinity and specificity for target molecules, including proteins, peptides, metal ions, small molecules, and cancer cells, due to their unique three-dimensional folding [23,25,27]. They are considered targeted therapies (smart drugs) with low toxicity that selectively control cancer cell progression by inhibiting cell surface proteins [23,27,29]. Their selection occurs in vitro through an amplification process called systematic evolution of ligands by exponential enrichment (SELEX) [23,25]. Since the establishment of SELEX in 1990, many aptamers have been generated against a variety of targets, including small chemical compounds targeting large multi-domain proteins [23,25]. To date, there are 11 aptamers in clinical trials for the treatment various diseases, such as inflammation and cancer, including PCa [23].

The selection of an integrin α6β4-specific DNA aptamer (IDA) to block the α6β4 integrin/laminin-332 interaction was recently reported by Berg et al. [23]. IDA inhibits the adhesion of PC3 PCa cells to laminin-332, with a resulting IC_50_ of 149 nmol/L [23]. The binding of IDA to PC3 cells depends on α6 integrins, such as the α6β4 integrin, which opens the possibility to block interactions between the α6β1 integrin with other laminins as well [23]. In addition to IDA’s function as an inhibitor, this aptamer can be used as a biomarker for cancer cells with high expression of α6 integrins, such as epithelial or thyroid carcinomas [23]. Also, the internalization of IDA could make the direct delivery of drugs to cancer cells overexpressing the α6β4 integrin feasible. Several aptamer-drug conjugates have been published involving siRNA, microRNA, toxins, and functionalized nanoparticles [22,25,26].

## 6. d-Amino Acid Inhibits α5β1 Integrin

The endothelial cell α5β1 integrin fibronectin receptor can recognize single ligands, and has been shown to play a role in metastatic invasion during extravasation and angiogenesis [30,31,32,33,34]. For this reason, the development of a potent non-toxic inhibitor of α5β1 integrin could prevent invasion in human PCa cell lines and reduce metastatic progression [30].

### Ac-PhScN-NH2 (PhScN)

Peptide pharmaceuticals have been identified as important therapeutic compounds for cancer because of their low toxicity and high specificity [30]. The PHSCN peptide (Ac-PHSCN-NH2), which inhibits the activated form of α5β1 integrin, prevents invasion and metastasis [30,35]. PHSCN has been shown to be effective against breast cancer cells in a Phase I clinical trial [30]. A recent study has shown that the PhScN peptide (Ac-PhScN-NH2), a PHSCN derivative, is 4–5 times more potent than PHSCN as an inhibitor of the α5β1 integrin in DU145 and PC3 PCa cell lines [30,35]. Interestingly, the inhibitory mechanism of action of both PHSCN and PhScN is based on a non-covalent and disulfide bond formation between the peptides and the α5β1 integrin binding site [30]. PhScN demonstrated efficient inhibition of lung vasculature extravasation and colonization by DU145 and PC3 cells in vivo [30,35]. These results suggest that PhScN may be an effective therapy for reducing PCa progression.

Peptide pharmaceuticals with simple structures, such as PhScN, have several advantages compared to other therapeutic compounds, including high potency and specificity. Their small size allows them to penetrate tissues that may not be reached by larger molecules. However, a major disadvantage of these agents is their rapid degradation by proteolytic enzymes, such as aminopeptidases and carboxypeptidases [30]. Nevertheless, *N*-acetylation and *C*-amidation makes PhScN resistant to proteolytic degradation [30,31,35].

## 7. Snake Venom Inhibits Various Integrins

The expression of the αvβ1, αvβ5, αvβ3, and α2β3 integrins correlates with the ability of cells to adhere and migrate to ECM proteins in vitro, as well as the aggressiveness of the cancer cell lines in vivo [36,37,38]. Thus, inhibition of these integrins may prove to be an attractive method to prevent metastasis.

### Contortrostatin

Contortrostatin (CN) is a non-toxic component in the venom of the southern Copperhead snake that functions as a platelet integrin antagonist, thereby inhibiting their aggregation [36,37,38,39]. This effect allows for the efficient spread of the venom components and it has also been shown to have antitumor activities against various cancers, including melanoma and breast cancer [36,37,38,39,40]. Previous studies have shown that CN alone and in combination with docetaxel can affect the function of several integrins, including αvβ1, αvβ3 and αvβ5, and αIIβ3, which are expressed in PC3 and CWR22 PCa cell lines [36]. The PC3 cell line has been shown to express αvβ1 and αvβ5 integrins, but not αvβ3 integrin [36,38]. In vitro adhesion assays revealed that PC3 cells can bind to immobilized fibronectin, vitronectin, and laminin [36]. CN inhibited adhesion to fibronectin and vitronectin dose-dependently in this cell line, but it did not have a significant effect on binding to laminin [36,37,38,39,40].

CN has been shown to inhibit PC3 cell growth, detachment, and migration [36]. These results demonstrated that different integrins in PC3 cells, such as αvβ1 and αvβ5, can be inhibited by this compound [36,37]. These effects are additive to the inhibitory effect caused by docetaxel treatment when both compounds are administered in combination [36]. Docetaxel, a microtubule inhibitor, is currently the primary chemotherapy drug used against PCa [36]. Interestingly, combination therapy with CN and docetaxel has been shown to have a synergistic effect resulting in greater inhibition of PCa cells division [36]. Furthermore, one of the limitations of CN is that it cannot be produced in large quantities because it is obtained from snake venom. Nevertheless, new formulations of CN are improving the pharmacokinetic profile of the drug, such as liposomal formulation of CN (LCN). This dramatically increase the half-life of LCN over the original CN and allows better drug release inside tumor cells [36,37,38,39,40,41].

## 8. Non-Peptides and Peptides Inhibitors

The expression of αvβ3 and αvβ5 integrins in advanced PCa is upregulated compared to normal prostate cells because they are functional integrins with metastasis initiating properties [42]. The most frequent metastatic site for PCa is the bone marrow and it has been shown that fully functional expression of both αvβ3 or αvβ5 integrin promotes PCa growth in bone [42]. Previous studies have shown that the use of an arginine-glycine-aspartic acid (RGD) amino acid sequence receptor can block αvβ3 and αvβ5 integrin as well as progression of bone metastasis in PCa [42,43,44,45,46].

### 8.1. GLPG0187

GLPG0187 is a non-peptide RGD integrin receptor antagonist [42]. GLPG0187 have shown selectivity for the αvβ3 integrin with an IC_50_ value of 2.0 nM in PC3 PCa cells [42,43]. In vitro solid-phase integrin ligand-binding assays have demonstrated that GLPG0187 dose-dependently decreased cell adhesion and cell migration in PC3 PCa cells, which have tumor and metastasis ability [42,44]. Interestingly, proliferation of PC3 PCa cells after 48 and 72 h of treatment was significantly decreased, but no significant differences in cell death were found [42]. In addition to inhibiting αvβ3 integrin in tumor cells, GLPG0187 dose-dependently affect angiogenesis and osteoclastic bone resorption in vivo resulting in smaller prostate tumors. In vivo results have demonstrated that the progression of bone metastasis was significantly inhibited after 12 days of treatment and the tumor weight was decreased after 15 days of treatment with both 30 and 100 mg/kg per day [42]. These results have shown that in already existing bone metastasis the blockage of αvβ3 integrin was strongly inhibited by GLPG0187 [42].

### 8.2. MK-0429

MK-0429 is a non-peptide small molecule inhibitor of the αvβ3 integrin that has demonstrated potent inhibition of osteoclast formation and osteoclastic bone resorption in both preclinical and clinical studies [47]. A study was performed to evaluate the tolerability and safety of MK-0429 in patients with hormone refractory PCa (PCa that does not responds to treatment with hormones) with bone metastasis [47]. MK-0429 was used to measure a biochemical marker of bone turnover named Urine *N*-telopeptide of type 1 collagen (uNTx) [47]. Results showed that the administration of MK-0429 two times per day for 4 weeks significant reduces 43.4% of 200 mg uNTx in men [47]. In other words, the inhibition of αvβ3 integrin with MK-0429 in patients was well tolerated and there was evidence of reduction of osteoclast activity indicating a potential for clinical use in the treatment for PCa [47].

### 8.3. Cilengitide (EMD121974, NSC, 707544)

Cilengitide is a cyclic pentapeptide and RGD mimetic that selectively and competitively antagonizes ligand binding to αvβ3 and αvβ5 integrins in vitro [48]. In vitro studies have shown inhibition of proliferation and increased apoptosis of PCa cell lines resulting in decreased osteoclast activity tumor regression in cell culture [48]. In addition, in a phase I trial, Cilengitide has shown clinical activity as a competitive inhibitor of the αvβ3 and αvβ5 integrins at both low and high dose levels. This suggests no linear function in treatment dose-response [48,49,50]. Nevertheless, a phase II trial determined that Cilengitide was well tolerated, but there was no change in PSA levels, suggesting no detectable clinical activity [49]. These findings suggest that for a phase III trial there is the need to identify the best dose level of Cilengitide and a combination with conventional chemotherapy that can enhance the activity of this integrin inhibitor [48,49,50].

## 9. Summary

**Figure 1:**
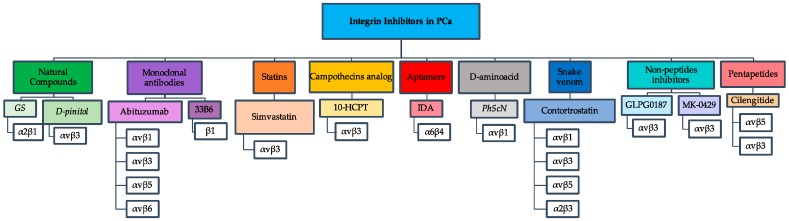
Representative flowchart of integrin inhibitors used against PCa. The flowchart depicts seven different groups of integrin inhibitors with their respective integrin receptors that were studied as drug targets in PCa.

In summary, the study of integrin inhibitors have helped to characterize integrin specificity (Figure 1) and have shed light on the multiple carcinogenic pathways that these inhibitors can control, such as regulators of cell survival, adhesion, migration, and proliferation. The study of integrin behavior, along with the development of compounds that work as integrin inhibitors, are important in order to develop novel and more effective therapies against PCa progression. Nevertheless, in addition to integrins and their inhibitors, it is necessary to continue to investigate the differences in the molecular composition of PCa cells (androgen-dependent and androgen-independent) in order to identify additional potential targets.

## 10. Conclusions

This review discusses the use of integrin inhibitors as anti-PCa treatments. The studies reviewed here have shown that targeting integrins and their subunits can affect diverse signaling pathways that are responsible for PCa progression. The development and discovery of new molecules, natural compounds, drugs, formulations, non-peptide, and peptide inhibitors that can target specific protein-ligand binding sites of interest in PCa remains a challenge. Collectively, the data provided supports the important pharmacological effects of each integrin inhibitor as anticancer agents. We classified integrin inhibitors as new active agents for PCa treatment and emphasized various aspects of their inhibitory properties, such as high specificity for their target and low toxicity. Nevertheless, it is important to mention that there is a need for additional research to investigate the biological role of integrins in bone microenvironments, because this is another potential target in the progression of metastatic PCa cells. This research will significantly contribute to a better understanding of the cellular behavior of integrins and their ECM ligands in different stages of PCa. On the other hand, it is important to know how to control integrins by targeting other biological pathways, such as cell metabolic pathways. A better understanding of how to inhibit integrins will drive this area of research forward and will enrich what is currently known about how to treat PCa cancers that are resistant to chemotherapy, will help in the development of new biomarkers that can be used for personalized therapy, and will improve actual chemotherapeutic strategies and help in the developments of new ones. In sum, targeting the biomolecular interactions between integrins and their inhibitors has great potential in PCa therapeutics.

## Figures and Tables

**Table 1:** Integrin expression in PC3, 22RV1 or CWR22 prostate cancer (PCa) cells. This table summarizes the alterations in well-known integrin receptors for collagen, fibrinogen, vitronectin, fibronectin, and laminin found in PC3, 22RRV1 or CWR22 PCa cell lines.

Ligand	Integrin	Effect	Cell Line Tested
*Collagen*	αvβ1	Upregulated	PC3, CWR22
	α2β1	Upregulated	PC3
*Fibrinogen*	αIIβ3	Upregulated	PC3, CWR22
	αvβ3	Upregulated	PC3, 22RV1, CWR22
*Vitronectin*	αvβ3	Upregulated	PC3, 22RV1, CWR22
	αvβ5	Upregulated	PC3, 22RV1, CWR22
*Fibronectin*	αvβ1	Upregulated	PC3, DU145
	α2β1	Upregulated	PC3, CWR22
*Laminin*	α6β4	Upregulated	PC3
